# Learning and working on an interprofessional training ward in neonatology improves interprofessional competencies

**DOI:** 10.3389/fmed.2025.1483551

**Published:** 2025-02-05

**Authors:** Hannah L. Schwarz, Christine Straub, Sebastian F. N. Bode, Nicole Ferschl, Christian Brickmann, Pascal O. Berberat, Marcus Krüger

**Affiliations:** ^1^Department of Neonatology, Muenchen Klinik gGmbH, Munich, Germany; ^2^Department of General Pediatrics, Adolescent Medicine and Neonatology, Medical Centre, Faculty of Medicine, University of Freiburg, Freiburg, Germany; ^3^Department of Pediatrics and Adolescent Medicine, Ulm University Medical Center, Ulm University, Ulm, Germany; ^4^Department of Pediatrics, School of Medicine, Technical University of Munich, Munich, Germany; ^5^Department of Clinical Medicine, TUM School of Medicine and Health, TUM Medical Education Center, Technical University of Munich, Munich, Germany

**Keywords:** interprofessional training ward, interprofessional learning, interprofessional practice, interprofessional competencies, interprofessionality in neonatology

## Abstract

**Introduction:**

Interprofessional education (IPE) is essential for healthcare professionals to prepare them for future interprofessional collaboration (IPC). Interprofessional training wards (ITWs) have been set up for IPE and results have been published. There are no published studies on ITWs in neonatology. We have designed and established the Interprofessional Training Ward in Neonatology (IPANEO) for nursing trainees (NT) and medical students (MS) in a neonatological intermediate care (IMC) ward. We report on the concept and the results with regard to the interprofessional competencies of the participants, including parent satisfaction.

**Methods:**

Supervision by medical and nursing learning facilitators, 2week blocks each with 2 NT (*n* = 30) and 2 MS (*n* = 23) in their final year, ward-in-ward concept, 3 patients cared for. Evaluation of the participants (pre/post) with the Interprofessional Socialisation and Valuing Scale (ISVS), the Interprofessional Collaboration Scale (ICS) with questions on IP communication, accommodation and isolation as well as with an IPANEO-specific evaluation (IPQ), an external evaluation with the “Observational Questionnaire for Learning Facilitators” (OQLF) and a “Questionnaire on Parent Satisfaction” (PSQ) (*n* = 33).

**Results:**

IPANEO participants showed significant increases in competencies in IP communication, accomodation and isolation (ICS), a better IP-collaboration and a higher role definition (IPANEO specific questionnaire). The ISVS 9A/B global scores increased. According to the self-assessment there were significant improvements in the external evaluation in all IP-categories (OQLF). The feedback from the parents was significantly positive (PSQ).

**Conclusion:**

Interprofessional learning and working on IPANEO had a positive impact on interprofessional competencies with high parent satisfaction.

## 1 Introduction

Interprofessional collaboration (IPC) is essential for a good patient-centered care in today’s healthcare system (1–3). In Germany, as in many other countries (4), an interprofessional (IP) training structure has not yet been established [(5), p. 26, (6), p. 17, (7)], although this has long been called for (inter)nationally [(8), p. 7, (9), (10), p. 3, (11), p. 17].

Work-based learning as interprofessional education (IPE) in the clinical setting has been shown to be particularly effective for subsequent IPC (12–18). One example of IP-based learning environments are interprofessional training wards (ITWs) (19). On ITWs, students from different healthcare professions learn from, with and about each other and are simultaneously responsible for the care of patients (19, 20). ITWs have mainly been established in adult medicine (11, 19, 21). Positive developments of participants of a rotation on an ITW with regard to professional role development, communication skills and IP competencies such as socialization and teamwork skills have been demonstrated (13–18). Long-term effects have been confirmed (22, 23). In addition, patient satisfaction is high and the cost-effectiveness of ITWs has been demonstrated (13–18, 24). To date, there are no accessible comparable studies that include self-assessment, external assessment of IP skills by qualified learning facilitators and patient or parent satisfaction in pediatrics (7, 17) and no publications on ITWs in neonatology (25). The special, sensitive patient cohort of premature and newborn infants entails a high degree of complexity in interaction and social structures and therefore places high demands on interprofessionality (25, 26). This requires precise coordination of interprofessional cooperation between medical staff, e.g., in the form of the concept of “minimal or optimal handling,” the reduction of unnecessary, stressful contact in order to minimize stress in premature or newborn babies (61). In addition, individualized communication with the parents that is appropriate to the particular life situation is necessary (27–29). The influence of individual experience and emotions on IP learning has been investigated (30). To date, this is a medical professional field that is not covered in great detail in medical degree programs in Germany (62), as in many other countries (31). As a result, it can be observed that medical doctors in the field of neonatology are increasingly dependent on the expertise of experienced nurses and interprofessional collaboration (26, 31). Informal learning by medical doctors from nurses has been reported (32) and the appreciation for integration into a kind of community of practice that nursing teams form has been demonstrated (33).

As a transfer project of the first Pediatric Interprofessional Training Ward in Germany (IPAPAED, Freiburg with funding from the Robert Bosch Stiftung), the Interprofessional Training Ward in Neonatology (IPANEO) was established at the “Muenchen Klinik Schwabing” on a neonatological IMC (intermediate care) ward, a neonatology unit of the highest level of care, in 2019. The IPANEO at the pediatric clinic of “Muenchen Klinik gGmbH” is therefore a learning unit based on the concept of the IPAPAED Freiburg (34).

The aim of the study was to evaluate whether participation in IPANEO leads to measurable improvements in participants’ IP competencies and to understand whether IPANEO participants benefit from their experience. We report on the results in terms of interprofessional competencies after a rotation on an ITW.

## 2 Methods

### 2.1 Interprofessional training ward in neonatology (IPANEO)

**Figure 1:**
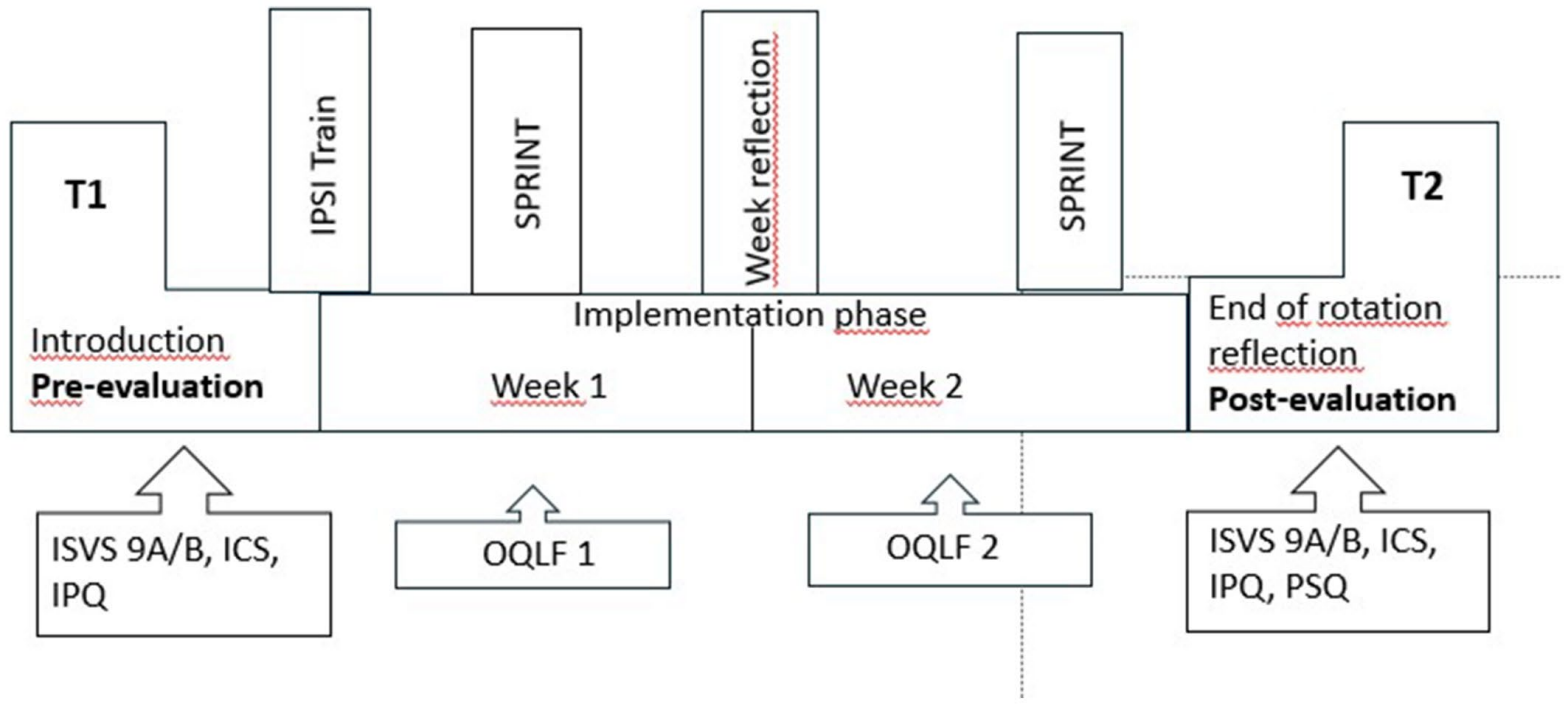
IPANEO- the concept (35). The two-week rotation is flanked by an introduction session and an end-of-rotation reflection. Pre- and post-evaluations include the ICS, the ISVS 9A/B, the OQLF, the IPQ and the PSQ. T1: ICS, Interprofessional collaboration scale, ISVS, Interprofessional collaboration and valuing scale. Week 1/2: OQLF, Observational questionnaire for learning facilitators. T2: IPQ, IPANEO specific questionnaire, PSQ, Parent specific questionnaire.

The interprofessional team on the IPANEO consists of two NT and two MS. The trainees work alternately in the early and late shift and, with the support of the team at the ward (ward-in-ward concept), also cares for the IPANEO patients before and after the daily IPANEO time. The interprofessional working time on the IPANEO starts at 08:00 a.m. with the arrival of the MS and the nursing and medical learning facilitators (LF) and ends at 04:00 p.m. At night and at weekends, the patients are cared for by the regular ward team. Interprofessional simulation (IPSI) on CPR/resuscitation is included in the two-week course (35) (Figure 1). A group reflection (36) is held daily at 01:00 p.m., followed by a “SPRINT- Speed InterProfessional PeeR TeachIng NeonaTology,” a short interprofessional peer-teaching unit [see “SIESTA,” (37)], which is integrated into the daily routine twice a week (from 01:30 p.m.; Figure 1). Learning facilitation and guidance follows an internal curriculum (6, 38, 39), which includes reflection on roles and responsibilities, team communication and professional identity (40). Structured concepts for the ward on pocket cards and a selection of patients with clearly defined clinical pictures also provide a framework (35).

### 2.2 Study design and cohort

Prospective, non-randomized, quasi-experimental study with pre- and post-questionnaires (T1, T2) before and after IPANEO, including assessment questionnaires on self-perception and external assessment as well as parent satisfaction (T2). The study population comprises 23 final-year MS of a six-year medical school program and 30 NT in their 2nd or 3rd year of training of a three-year nursing degree (a non-university degree in Germany) (total *n* = 53).

### 2.3 Data collection

All IPANEO participants from November 2019 to March 2022 (20 rotations) were included. The parent questionnaires were collected between October 2019 and December 2020 (*n* = 33). Participation was voluntary and participants provided written consent.

### 2.4 Quantitative methodology

The outcome measures were recorded using the ISVS - Interprofessional Socialisation and Valuing Scale [(41), p. 171ff], the ICS - Interprofessional Collaboration Scale (42) and, in addition, the IPANEO-specific questionnaire (IPQ) - a questionnaire created individually for Neonatology Schwabing [see (35)]. In addition, the data from the “Observational Questionnaire for Learning Facilitators” (OQLF) and the “Parent Satisfaction Questionnaire” (PSQ) were analysed (IPANEO specific questionnaires). The paper-based pre (T1) and post (T2) questionnaires were completed on the introductory day and on the last day. Only the PSQ and the second part of the IPQ (11–30) were only collected at T2.

#### 2.4.1 ISVS 9 set A and set B (*n* = 51)

The two short, 9-item equivalent forms of the ISVS have been applied, each subscale reflects key concepts of IP practice (41). The ISVS versions for IPANEO were adopted with the transfer of the IPAPAED, translated from English, and scientifically reviewed and validated (35). The ISVS was adopted with the transfer of the IPAPAED, translated from English, and scientifically reviewed and validated (35). 18 items measuring beliefs, attitudes and behaviors in relation to interprofessional relationships, collaboration and socialization were rated on a 7-point Likert scale from 6 (fully agree) to 0 (fully disagree) (Set A/B: 9 items each). The evaluation was based on the global scores and complemented by the assessment of the individual questions.

#### 2.4.2 ICS medicine (*n* = 22) and ICS nursing (*n* = 29)

Perceptions of communication, isolation and accomodation were measured in a 13-point survey. A rating from “1 = strongly disagree” to “4 = strongly agree” could be given. The three categories as well as the individual questions were evaluated in order to identify the most significant increases (communication, accommodation) or decreases (isolation).

#### 2.4.3 IPANEO specific questionnaire (*n* = 51)

The IPANEO specific questionnaire was adopted from the specially developed IPAPAED questionnaire (35) during the transfer from Freiburg and adapted for neonatology. Participants were able to select within a categorization from 1 (best possible) to 5 (7 items). This questionnaire includes demographic data, a project-specific evaluation as well as elements of communication, role definition and collaboration.

#### 2.4.4 IPANEO observational questionnaire for learning facilitators (*n* = 62)

An “observational questionnaire for learning facilitators” (transfer from Freiburg (39)) developed to assess the participating learners was evaluated interprofessionally by the nursing and medical learning facilitators during the 2 weeks (*n* = 117 observational questionnaires, Likert scale 1 to 3). For the calculations, values from the first week of implementation (initial assessment) were compared with the values from the second week (final assessment) [subdivided into IP communication (4 items), IP collaboration (5 items), IP role definition (3 items)].

#### 2.4.5 Parent satisfaction questionnaire (*n* = 29)

This questionnaire was transferred from IPAPAED Freiburg including general aspects of care and rating of the IPANEO (17). Parents used a Likert scale (1 to 4/ 1 to 5) to rate the care of their premature or newborn baby by the respective professional group and the interprofessional cooperation of the team. The length of stay on the ward and the gestational age (28–42 weeks’ gestation) of the premature/newborn baby were also documented.

### 2.5 Data analysis and statistics

Statistical calculation and data analysis in GraphPad (version 10) with the Wilcoxon-signed-rank-test (T1, T2) and the Mann–Whitney-U-test for the post-data (T2) of the IPQ (11–30). The median (m) and the *p*-value [(p), two-sided] are visualized as dominant values. Descriptive measures [median (m) in the confidence interval (CI), mean (me), standard deviation (SD)] were also used.

### 2.6 Ethics

The concept and implementation of IPANEO as well as the evaluation were approved by “München Klinik gGmbH.” All participants gave their written consent to complete the questionnaires and to be contacted by email and agreed to the publication of the anonymised data.

## 3 Results

All 53 IPANEO participants from November 2019 to March 2022 were included in the study. NT and MS were comparably represented in both groups. 86% of participants were female, all male participants were MS. The participants were on average 23 years old (18–33 years). Due to missing questionnaires all but 2 participants were included in the analysis resulting in a response rate of 96%. The age of the premature/newborn babies was at an average of 35–38 weeks during the period of care on the IPANEO (me = 5.2, SD = 1.0; PSQ 11) and the average length of stay on the ward was 8 days (me = 7.8, SD = 5.3; PSQ 9).

### 3.1 Quantitative evaluation

#### 3.1.1 Self-assessment

##### 3.1.1.1 High development of interprofessional socialization and valuing (ISVS)

**Figure 2:**
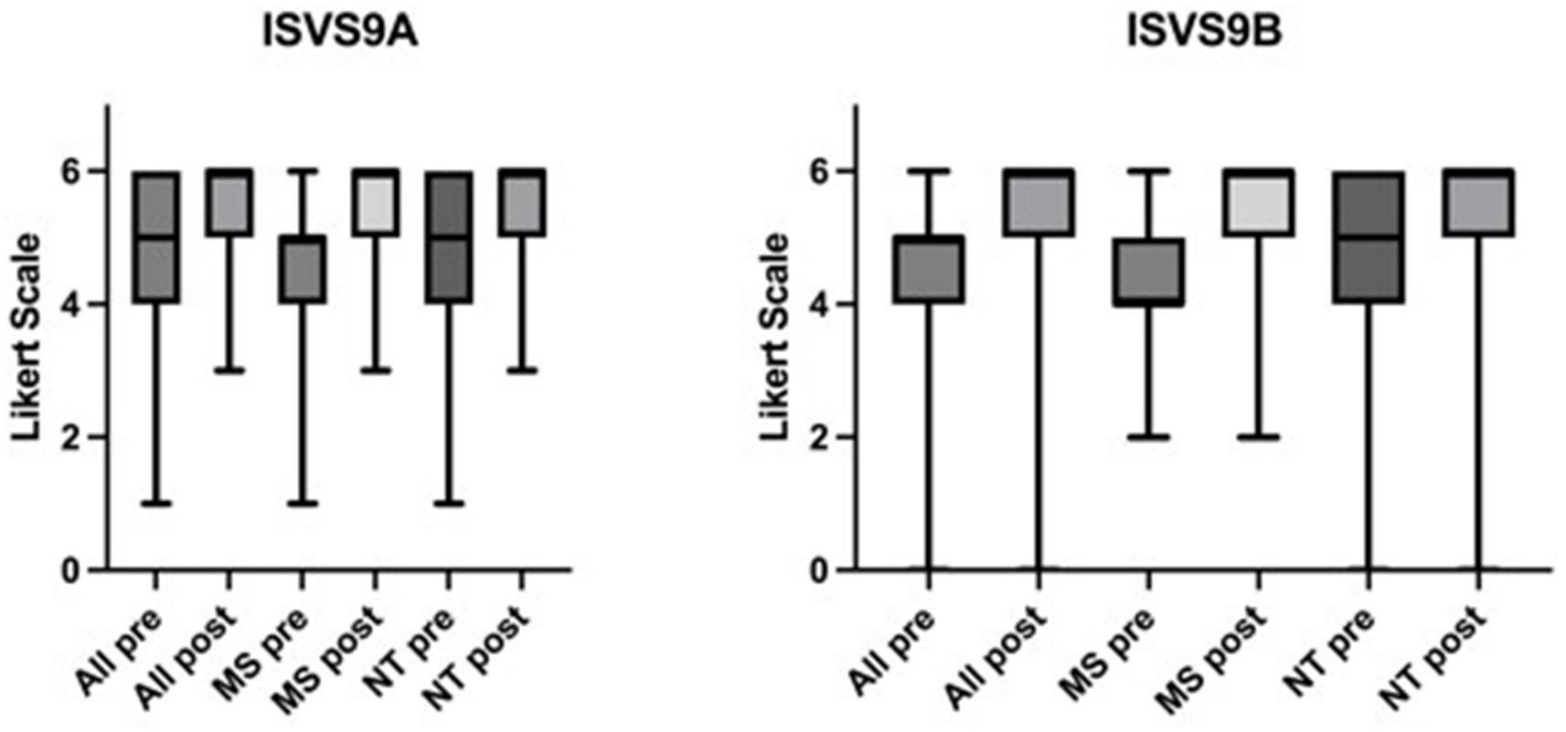
Significant changes in the ISVS 9A/B global scores. Scale from 0 to 6 on a Likert scale. Higher numbers indicate an increase in competencies. Pre =T1, post = T2. ISVS 9A/B-All, MS, NT, *p* < 0.001, *n*=459. ISVS 9A/B: Interprofessional Socialisation and Valuing Scale, 9- Item Equivalent versions. MS: edited by MS, NT: edited by NT.

The global scores of the ISVS 9A/B increased in both professional groups (PGs) (Figure 2). After the two-week IPANEO rotation the participants rated their competencies significantly higher in all IP categories, e.g., they stated an increase in the assumption of responsibility (*m* = 4 “agree,” pre; *m* = 6 “fully agree,” post; *p* < 0.0001, ISVS 9B-All, 7) and independence (*m* = 3 “partially agree,” pre; *m* = 6 “fully agree,” post, *p* < 0.0001, ISVS 9B-All, 2). According to their self- assessment all participants developed a significantly greater awareness of one’s own role in the team (*m* = 4 “agree,” pre; *m* = 6 “fully agree,” post; *p* < 0.0001, ISVS 9B-All, 1).

**Figure 3:**
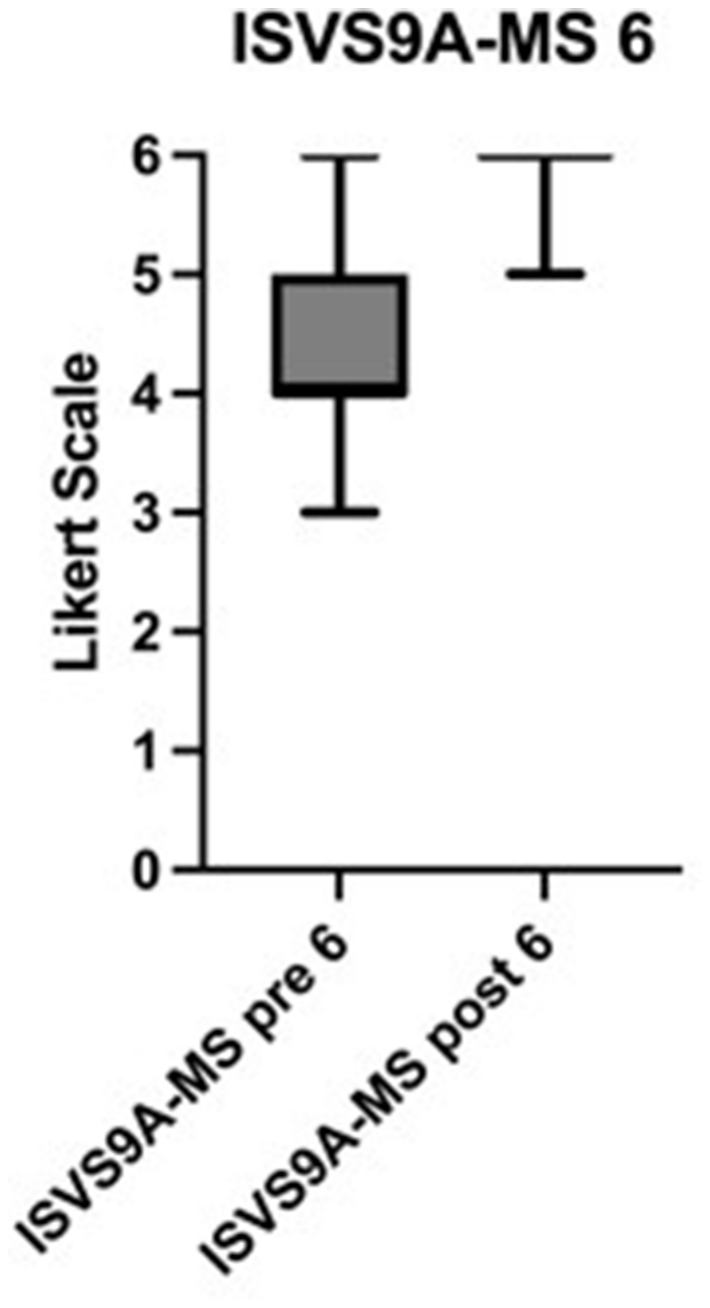
Awareness of nursing in a team. Significant changes in the ISVS 9A-MS 6 score in medical students. Scale from 0 to 6 on a Likert scale. Higher numbers indicate a significantly higher awareness of nursing in a team. Pre = T1, post = T2, *p* < 0.0001, *n*= 198. ISVS 9A: Interprofessional Socialisation and Valuing Scale, 9-Item Equivalent version. MS: edited by MS.

Both PGs showed a significant increase in the appreciation of how important it is to integrate families as members of the team (*m* = 5 “strongly agree,” pre; *m* = 6 “fully agree,” post; *p* < 0.0001, ISVS 9A-All, 7). In particular the NT developed a significantly higher understanding of involving patients in participatory decision-making in the context of their healthcare (*m* = 4 “agree,” pre; *m* = 6 “fully agree,” post; *p* < 0.0001; ISVS 9A-NT, 8). Likewise all participants favored working in an interprofessional team at T2 (*m* = 5 “strongly agree,” post; *p* < 0.0001; ISVS 9B-All, 3). Again, the highest significant increase in the commitment with interprofessional practice was found in the group of the NT (*m* = 4 “agree,” pre; *m* = 6 “fully agree,” post; *p* < 0.0001; ISVS 9A-NT, 2). MS reported to have acquired a significantly higher awareness of the role of nursing in a team through participation in and practical performance of nursing activities (*m* = 4 “agree,” pre, *m* = 6 “fully agree,” post, *p* < 0.0001; ISVS 9A-MS, Figure 3).

##### 3.1.1.2 Improvement of interprofessional cooperation (ICS)

The ICS is categorized in the dimensions communication, accommodation and isolation. In all three ICS categories the medians remained at a constant level. Significant increases were found in the following questions: Prior to participation the PGs had different treatment conceptions (*m* = 2 “disagree,” pre; *p* < 0.0001, ICS-NT, 4) and differences of opinion often remained unresolved (*m* = 3 “agree,” pre; *p* < 0.0001, ICS-MS, 11). These perceptions changed significantly to positive assessments after the IPANEO (*m* = 3 “agree,” post, ICS-NT, 4, *m* = 2 “disagree,” post, ICS-MS, 11).

##### 3.1.1.3 Increasing importance of interprofessional collaboration (IPQ)

After the IPANEO the importance of IP communication for patient care was rated very highly by the participants (*m* = 1 “very high importance,” pre, post, no significant difference between the PGs, post; IPQ-All, 8) and satisfaction with the feedback culture increased significantly (*m* = 3 “partly/partly,” pre; *m* = 2 “satisfied,” post; *p* < 0.0001, no significant difference between the PGs, post; IPQ-All, 10). Concerning the IP-collaboration the motivation to utilize the support of the other PG increased (*m* = 2, “high,” pre; *m* = 1 “very high,” post; *p*-value <0.0001; significant difference between the PGs, post; *p* = 0.015, IPQ-All, 8).

#### 3.1.2 External assessment

##### 3.1.2.1 High assessment by the learning facilitators (OQLF)

In the external evaluation by the learning facilitators there were significant increases in the ability to communicate with other PGs and parents (*m* = 2, “with help,” pre; *m* = 1, “confident,” post; *p* < 0.0001, OQLF, 10–13). In addition a significant increase in the definition of one’s own role as well as the role of the other PG was found (*m* = 2, “with help,” pre; *m* = 1, “confident” post; *p* < 0.0001, OQLF, 14–16). Concerning IP-collaboration the participants improved the “interdisciplinary cooperation with members of other professional groups” significantly and reached an evaluation result of a “safe interprofessional cooperation” (*m* = 1 “safe,” post; *p* < 0.0001; OQLF, 1–5).

##### 3.1.2.2 Parents’ satisfaction with the treatment (PSQ)

The results of the parents questioning confirm good care from the IPANEO team, which had a positive effect on the child’s treatment (*m* = 1, “very good,” CI = 0.96; PSQ, 2, 3). The parents stated that they had received all important information about the clinical course (*m* = 1, “definitely,” CI = 0.96; PSQ, 5). The treatment team of students and trainees was perceived by the parents as an interprofessional team (*m* = 1, “very good,” CI = 0.96; PSQ, 4). If necessary, 98% of the parents surveyed would agree to repeat treatment on IPANEO (*m* = 1, “definitely,” CI = 0.98, PSQ, 6).

Based on these results a rotation on a neonatological ITW appears to have a positive effect on IP competencies and interprofessional training on an IMC at a neonatological (university) hospital appears to be feasible in terms of learning success.

## 4 Discussion

This study is the first to report on the outcomes of a voluntary rotation on an ITW in neonatology, including parent satisfaction and supervision by board-certified professionals. The importance of a clear structure (see Figure 1) in the changing context of professional IP training was highlighted (43). In order to initiate the lifelong learning process of competence development, the participants were actively encouraged to form an interprofessional team and take responsibility through the teaching concept (44, 45). As a result, they recognized that treatment success for patients can be achieved in an IP team (35). After ITWs in internal medicine and surgery improved interprofessional collaboration and teamwork as well as typical dynamic group development processes were reported: A significant increase in the assumption of responsibility and independence, information sharing as well as conflict resolution was found (13, 19–21, 46). As defined in the CanMEDs concept, one of the main tasks of physicians is to be a “member of a team” (47). Our results support the development of participants into team players: We show a significant increase in well-being in participatory decision-making within the team and with patients (48, 49). Extensive group reflection was conducted in line with the discussion of social constructive theory and interprofessional learning (36). The learning facilitators encouraged a culture of speaking up and listening, creating a “safe place with space for learning” (36).

**Figure 4:**
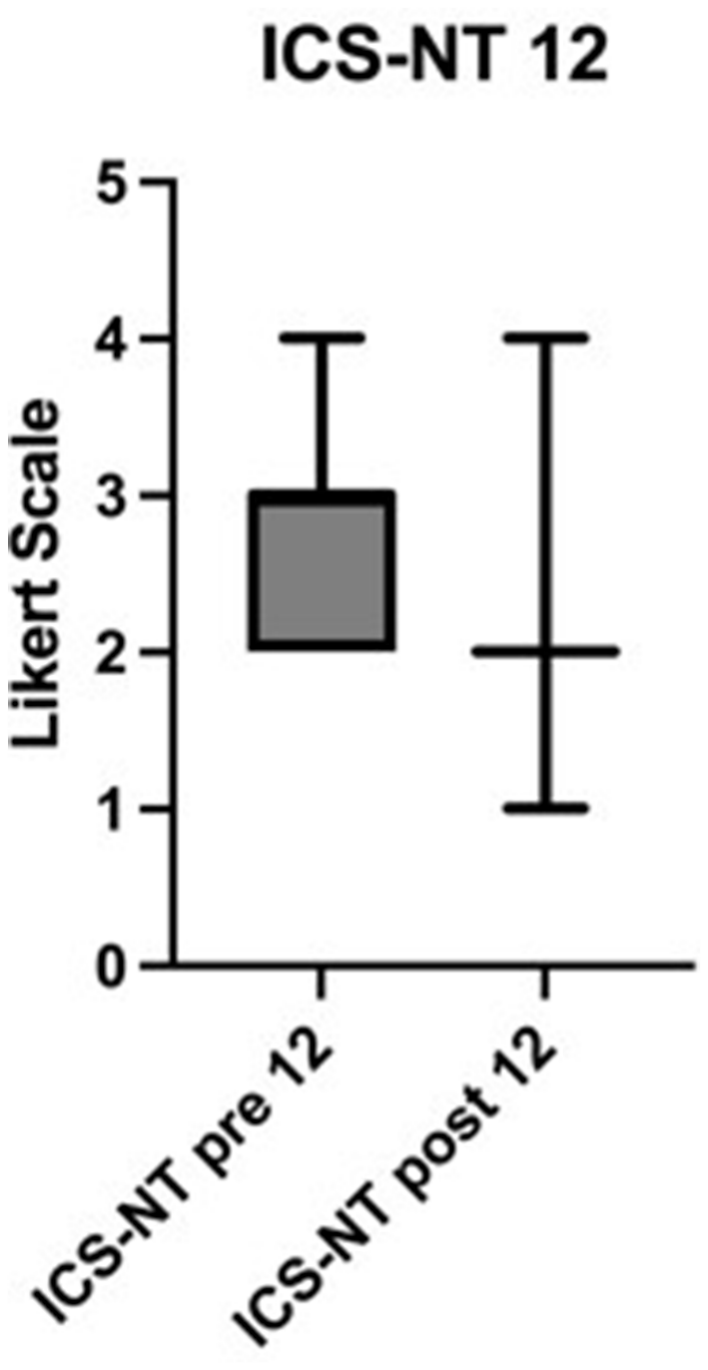
Shift in the role image of nursing care. Significant change in the ICS-NT 12 score in nursing trainees. Scale from 1 to 4 on a Likert Scale. Lower numbers indicate a higher role image (compared to physicians). Pre = T1, post = T2, *p* < 0.0001, *n* = 150. ICS-NT: Interprofessional collaboration scale, NT: edited by NT.

Profession-specific differences in the acquisition of interprofessional competencies have been reported (11, 20). In the pre-evaluation the participants rated physicians’ activities and decisions more important (*m* = 3 “agree,” pre; *p* < 0.0001, ICS-NT, 12, Figure 4), due to a reluctance to discuss new treatment methods (*m* = 3 “agree,” pre, post; *p* < 0.0001, ICS-NT, 7) and to ask for the opinion of the other PG (*m* = 2 “disagree” pre, post; *p* < 0.0001, ICS-NT, 8). After participation, the answers shifted significantly in the direction of a role image of both professions that was perceived as equal (*m* = 2 “disagree,” post, ICS-NT, 12, Figure 4). Likewise NT rate their own profession as less equal than that of their medical colleagues (50, 51). Possible solutions to this imbalance appear to be a reduction in hierarchies, as well as a change in task division and areas of responsibility (50, 51). However, this requires the cooperation and collaboration of all professions involved [(52), p. 19ff]. The historically shaped hierarchy in the healthcare system ascribes a key role to the physician’s profession, even in times of change [(5), p. 182]. A significant increase in the appreciation of the nursing profession has been shown (53, 54, 63). It is therefore understandable that NTs in particular are emerging as future multipliers for IP collaboration (51, 55). This indicates that different professional groups benefit in different ways from a rotation on an ITW (34).

As with many IPE concepts, assessing the impact remains a challenge (23, 56). A strength of this study is encountering this challenge with a comprehensive evaluation (57). Limitations are the conduction of the study in a neonatological context only, the rather small sample size and the lack of a qualitative data analysis with regard to interprofessional competencies and a control group. In addition, the timing of the data collection immediately before and after the intervention means that only short-term effects can be assumed with the results presented.

## 5 Conclusion

Future research on IPE should include qualitative analyses in order to investigate the background and motives for the aforementioned changes in behavior as well as the increase in competence and examine the long-term effects more closely. Repeated formal, objective evaluations of IPE participants and a control group without interprofessional intervention is desirable. In addition, the effects on IPC should be recorded by evaluating the staff of the wards or clinics where IPE takes place. The aim should be to include other professional groups as trainees in healthcare and pediatric nursing, physiotherapy and occupational therapy, students of medicine, pharmacy, midwifery and other PGs (“Scandinavian model”) to participate in interprofessional training (58) and to implement IPE as an integral part of the curricula at all training levels in the long term (3, 59, 60).

## Data Availability

The original contributions presented in the study are included in the article/supplementary material, further inquiries can be directed to the corresponding author.
